# Relationships Between Immune‐Inflammatory Features and Social Cognitive Impairments in Patients With Schizophrenia Spectrum Disorders: A Systematic Review

**DOI:** 10.1002/brb3.70384

**Published:** 2025-03-30

**Authors:** Alexandre Carpentier, Dimitrios Zampetas, Alexandre Durand, Mickael Naassila, Marie‐Cécile Bralet

**Affiliations:** ^1^ Service Pathologies Résistantes (SPR), Pôle Ressource Évaluation en Réhabilitation PsychoSociale (PRERPS) Centre Hospitalier Isarien––EPSM de l'Oise Clermont de l'Oise France; ^2^ Université Picardie Jules Verne Amiens France; ^3^ Institut de Psychiatrie (CNRS GDR 3557) Paris France; ^4^ INSERM UMR 1247––Groupe de Recherche sur l'Alcool et les Pharmacodépendances (GRAP) Université de Picardie Jules Verne, Centre Universitaire de Recherche en Santé Amiens France; ^5^ Service CRISALID‐HDF, Centre support de réhabilitation psychosociale et de remédiation cognitive, PRERPS Centre Hospitalier Isarien––EPSM de l'Oise Clermont de l'Oise France

**Keywords:** inflammatory markers, schizophrenia spectrum disorders, social cognition

## Abstract

**Introduction:**

Patients with schizophrenia spectrum disorders (SSD), particularly patients with schizophrenia, have social cognitive impairments characterized by difficulties in emotion recognition, the ability to attribute mental states, explaining the causes of events, and identifying and utilizing social cues. These impairments appear from early life and are associated with poor functional and social prognosis. The origin of these impairments is not fully understood. The inflammatory hypothesis is one of the pathophysiological hypotheses of schizophrenia. Inflammatory marker abnormalities are also present in the early stages of schizophrenia and are associated with neuronal degeneration. Following our main hypothesis, the aim of this work was to conduct a review to explore the relationship between social cognition and inflammatory markers in SSD.

**Methods:**

The review included original studies reporting measures of social cognition and plasma levels of inflammatory markers in patients with SSD using the Pubmed, PsycINFO, and Embase databases. The PRISMA methodology was followed.

**Results:**

Eleven studies were selected and analyzed. They showed significant correlations between plasma cytokine levels and theory of mind and facial emotion recognition abilities.

**Conclusion:**

The correlations do not seem to be specific to social cognitive impairments, but our results support the hypothesis of a link between pro‐ and anti‐inflammatory markers and cognition in SSD. In the future, other studies should be conducted to clarify this link from a diagnostic and therapeutic perspective: identification of inflammatory trait factors and patient subgroups and personalized anti‐inflammatory therapies.

## Graphical abstract

There is evidence indicating a link between inflammatory markers and theory of mind and facial emotion recognition impairments in SSD. Personalized treatment approaches targeting inflammatory profiles could offer new therapeutic avenues to address social cognitive impairments in subgroups of patients with SSD.

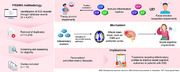

## 1 Introduction

Schizophrenia spectrum disorders (SSDs) encompass a range of psychiatric symptoms characterized by delusions, hallucinations, disorganized thinking, and negative symptoms, with varying severity and duration. This spectrum notably includes schizoaffective disorder, schizophreniform disorder, and, most prominently, schizophrenia, which remains the most extensively studied condition in the literature. SSD and particularly schizophrenia are correlated with a significant impact on psychosocial functioning (Handest et al. 2023). However, numerous studies have shown that functional impairments in patients with schizophrenia are mainly related to cognitive impairments (Harvey et al. 2022; Kharawala et al. 2021; McCutcheon et al. 2023). The most common cognitive impairments occur in neurocognition (executive function, processing speed, memory, attention, verbal fluency, problem‐solving) and social cognition (Fett et al. 2011). Among these impairments found in schizophrenia, social cognitive impairments are most closely associated with functional prognosis and represent a predominant factor influencing recovery (Green et al. 2015). Social cognition is considered a psychological multidimensional construct that includes the following five core domains: emotional processing (including facial emotion recognition [FER], expression, and prosody), theory of mind (ToM), attribution style, social perception, and social knowledge (Varo et al.,^2019^). In patients with schizophrenia, the most impaired social cognitive domains consist of FER (Gao et al. 2021), emotional processing prosody (Lin et al. 2018; Zhao et al. 2023), and ToM (van Neerven et al. 2021). Moreover, according to recent studies, ToM and FER impairments observed in social cognition among individuals with schizophrenia are considered trait impairments (Wu et al. 2023). These impairments persist across different contexts and are not solely associated with positive or negative symptoms or psychotropic medication effects (McCutcheon et al. 2023). Furthermore, although the results are heterogeneous, patients with schizophrenia would show substantial impairments in attributional style, social perception, and social knowledge, associated with poor insight and outcome in different functional domains (Green et al. 2019; Kitoko et al. 2020; Vaskinn and Horan 2020). The origin of these cognitive impairments is not entirely clear. However, there are several models to explain the pathophysiology of schizophrenia. The most widely accepted pathophysiological model of schizophrenia is the neurodevelopmental model. The neurodevelopmental model identifies two key periods of vulnerability in neurodevelopment as follows: early life and adolescence. This concept is framed within the environmental double‐hit model, which suggests that the onset of the disease requires two distinct events. The first event, or “hit,” may involve the interplay of stress factors and genetic vulnerability factors affecting fetal brain development while the second occurs during adolescence, triggering the emergence of the disorder (Davis et al. 2016; Fatemi and Folsom 2009; Owen et al. 2011; Owen and O'Donovan 2017). This model is intertwined with the hypothesis of an immuno‐inflammatory cascade. According to this model, inflammatory processes activate microglia, triggering the release of pro‐inflammatory cytokines in the central nervous system, which can contribute to the pathophysiology of schizophrenia (Shebl 2024). Microglia can become sensitized by stimuli such as neurodegeneration, aging, or stress, leading to an exaggerated immune response to even minor triggers. Following priming, a secondary stimulus, such as mild systemic inflammation or stress, can result in microglial proliferation and heightened cytokine production. This cytokine cascade stimulates the hypothalamic–pituitary–adrenal (HPA) axis resulting in increased cortisol secretion. Chronic HPA activation can dysregulate feedback mechanisms, leading to either hypercortisolism or hypocortisolism, both of which are observed in patients with schizophrenia (Farcas et al. 2023). Other systemic inflammatory markers, such as C‐reactive protein (CRP), are also elevated in response to the cytokine cascade, particularly due to increased secretion of Interleukin‐6 (IL‐6), which is associated with cognitive impairments in patients with schizophrenia (Inova et al. 2025). However, CRP and cortisol are limited by their lack of specificity, with CRP reflecting generalized inflammation and cortisol subject to considerable interindividual variability (Hawley et al. 2016; Orsolini et al. 2018). This is why most research focuses on measuring cytokines, the key inflammatory molecules, as they provide a more precise measure of inflammation and help identify specific pathways such as IL‐6 and TNF‐α, which are directly linked to neuroinflammation and cognitive impairments in schizophrenia. This hypersensitivity and elevated cytokine release may partially explain exacerbations of behavioral disturbances and cognitive impairments in patients with schizophrenia and mediate illness relapse (Li et al. 2023; Zhuo et al. 2023). Indeed, drug‐naïve patients during the first episode of psychosis would have significantly higher IL‐6, Interleukin‐1β (IL‐1β), and tumor necrosis factor α (TNF‐α) plasma levels compared with healthy controls (Zhang et al. 2021). Moreover, higher plasma levels of pro‐inflammatory cytokines IL‐6 and IL‐4 would also be present in ultra‐high‐risk psychosis individuals compared with healthy controls (Halstead et al. 2023).

The existence of commonalities seems to appear between social cognition and immuno‐inflammatory markers. Indeed, social cognitive impairments would be associated with poor prognosis and functional outcome (Luo et al. 2021). Moreover, they appear early in the course of development and are present throughout the illness (Sheffield et al. 2018). Likewise, inflammatory markers tend to be increased early in the course of illness, correlated with poor outcomes and associated with neurocognitive impairments (Kogan et al. 2020). Although neurocognitive impairments seem to be correlated to increased secretion of pro‐inflammatory markers and social cognition is considered a mediator between neurocognitive functions and social functioning, the link between social cognition and immuno‐inflammatory markers in schizophrenia remains poorly explored in scientific literature as indicated in a recent review (Adraoui et al. 2023). Adraoui and colleagues provide a concise summary of the neurobiological abnormalities affecting brain networks that lead to social cognitive impairments in patients with schizophrenia. Indeed, social cognitive impairments are thought to result from the interaction between oxidative stress, neuroinflammation, and NMDA receptor hypofunction, which disrupt the white matter networks critical for the efficiency of social cognitive abilities. However, they did not precisely describe the cytokine variations observed specifically for each domain of social cognition. Following these observations, the aim of our work was to precise the relationships between inflammatory markers and social cognitive impairments in patients with SSD. Identifying specific inflammatory markers of social cognitive impairments in patients with SSD may provide a better understanding of the pathogenesis of these impairments and could enable a more personalized approach to patient care. Consequently, we conducted the first systematic review illustrating the relationships between inflammatory markers and the different domains of social cognition in patients with SSD, particularly schizophrenia.

## 2 Materials and Methods

### 2.1 Registration and Reporting

A systematic review and narrative synthesis were carried out according to the guidelines of Preferred Reporting Items for Systematic Reviews and Meta‐analysis (PRISMA) statement and registered on PROSPERO (CRD42024597755) (Page et al. 2021).

### 2.2 Databases and Search Terms

Using an extended range of data sources (MEDLINE, EMBASE, PsycINFO) and phrases to characterize the sample of interest (i.e., individuals diagnosed with SSD) and the phenomena of interest (i.e., immunoinflammation, social cognitive impairments), the search strategy covered trajectories of researching inflammation and social cognitive impairments in SSD. A comprehensive computerized search was performed using medical subject headings for articles (MeSH). Regarding inflammatory markers, we have chosen to study the most sensitive markers in the pathophysiology of SSD using the following search terms in their singular or plural form in the title, abstract, keywords, and text fields of the articles: “interleukins,” “cytokines,” and “inflammation.” The term «inflammation» was used for other articles including less sensitive or more uncommon inflammatory markers. Regarding social cognitive impairments, we used the general terms of social cognition and the specific terms of the subdomains recognized in the literature as follows: “social cognition,” “social cognitive processing,” “emotion processing,” “theory of mind,” “attributional bias,” “social perception,” and “social knowledge.” We also use the terms “cognitive impairments” and “cognitive disorders” in order not to omit potential articles in which social cognitive impairments were not mentioned in the title or the abstract. Finally, all these terms were combined with the following terms to describe the population: “schizophrenia,” “schizophrenia spectrum disorders,” and “psychotic disorders.”

### 2.3 Inclusion and Exclusion Criteria

We considered articles published in English in peer‐reviewed journals involving (a) individuals (age >18) meeting either ICD‐10 or DSM‐IV or DSM‐5 diagnostic criteria for SSDs and other psychotic disorders (confirmed through a validated structured diagnostic interview) and (b) studies including serum or plasma inflammatory markers and standardized neuropsychological assessment tools focusing on at least one domain of social cognition. To maximize the number of searched articles, the publication year was not limited.

As this review was focused on social cognitive impairments and plasma levels of inflammatory markers in patients with SSD, we excluded studies exploring only the link between inflammatory markers and neurocognitive impairments. Articles were also excluded if they were books, chapters, dissertations, theses, reviews, or opinion pieces. Reviews were excluded but their references were searched.

The initial search criteria were defined in accordance with PICO framework (Population, Intervention, Comparison, and Outcome) (Eriksen and Frandsen 2018). Titles and abstracts of retrieved citations were screened, selected full‐text articles were assessed for eligibility, and data were extracted.

### 2.4 Data Retrieval and Synthesis

**FIGURE 1:**
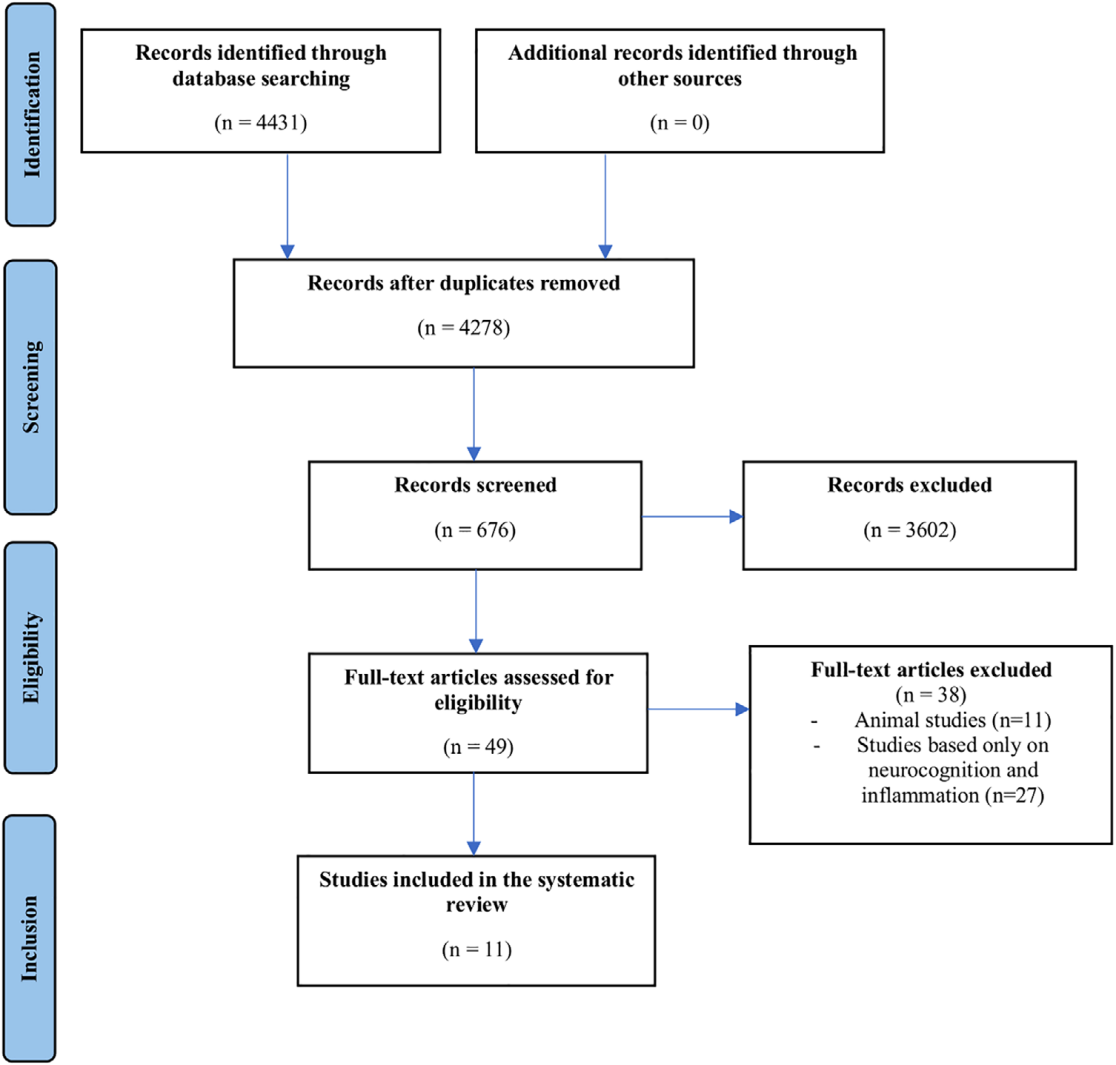
Overview of the study selection process for the systematic review, showing the identification, screening, eligibility, and inclusion of studies, with exclusions noted. A total of 4431 articles were retrieved, with 11 studies included after screening and eligibility assessment.

Studies retrieved from all databases were extracted to EndNote version X9.3.3 for Windows app–based citation manager, which was followed by the search of close and exact duplicates. All independent studies were exported to a Microsoft Excel spreadsheet to screen for further inclusion criteria. Two authors (D.Z. and A.C.) independently screened the titles and abstracts. Inconsistent decisions were discussed and solved with consensus. Finally, full text was reviewed, and data were independently extracted by two authors (D.Z. and A.C.) in order to assess study features, sample features (diagnoses et recruitment setting), and how the association of inflammatory markers and social cognitive impairments was described. The following data were extracted: (i) the number of participants, (ii) clinical characteristics of patients, (iii) the type of cytokine used among the included studies, (v) social cognitive assessment tools, and (vi) correlations between cytokines and social cognitive performance. The quality of this systematic review was evaluated using the AMSTAR 2 tool, which assesses 16 critical domains for systematic reviews (Shea et al. 2017). Key strengths of our review include a clearly defined research question, pre‐registration of the protocol on PROSPERO (CRD42024597755), and a comprehensive search strategy across major databases (PubMed, PsycINFO, and Embase). The inclusion and exclusion criteria were well‐documented, and a list of excluded studies with reasons for exclusion was provided (Figure 1). Furthermore, double‐screening and data extraction processes were employed to ensure rigor. The risk of bias in the included studies was assessed using a tailored approach based on established criteria, including elements from tools like ROBINS‐I. Overall, the risk of bias was determined to be moderate to high. Participant selection was generally appropriate, with clear diagnostic criteria based on DSM‐IV or DSM‐5, but some studies included heterogeneous populations or small sample sizes, which may introduce bias. The measurement of inflammatory markers was adequate but lacked consistent standardization across studies, potentially leading to variability in results. Social cognition was assessed using validated tools, resulting in a low risk of bias for outcome measurement. However, confounding variables such as duration of illness and antipsychotic treatment were not consistently accounted for, representing a significant source of potential bias. Additionally, some studies focused only on significant findings.

## 3 Results

A comprehensive search across all selected databases yielded a total of 4431 articles. After removing duplicates through initial screening based on titles and abstracts, a total of 676 articles remained for further evaluation and all these were included for title/abstract screening. The full‐text articles from this selection were thoroughly assessed for eligibility and quality, resulting in the final inclusion of 11 articles for the systematic review. These articles included 782 patients with a diagnosis of SSD according to the DSM‐IV or DSM‐5. The detailed selection process is presented in the PRISMA flow diagram in Figure 1 and characteristics of included studies are illustrated in Table 1.

**TABLE 1:** Details of included studies.

Authors and publication year	Inclusion criteria	Participants	Age	Social cognitive domain and assessment tools	Inflammatory markers	Summary of results
Walss‐Bass et al. (2013)	DSM‐IV‐TR diagnostic criteria for SZ or SZA	21 SZ/SZA with psychotic symptoms 39 SZ/SZA without psychotic symptoms 20 HC	SZ/SZA: 42.10 ± 10.84 HC: 39.65 ± 11.55	ToM (WRT)	IL‐6 IL‐10 TNF‐α IFN‐γ IL‐1β IL‐8 IL‐1RA IL‐2 sIL‐2R	Positive correlation between plasma IL‐1β, IL‐1RA, sIL‐2R, and IL‐10 levels and ToM impairment in SZ/SZA patients with psychotic symptoms
Dunne et al. (2017)	DSM‐IV‐TR diagnostic criteria for SZ or SZA Maintenance treatment with antipsychotics	21 SZ/SZA with psychotic symptoms 39 SZ/SZA without psychotic symptoms 20 HC	SZ/SZA: 42.10 ± 10.84 HC: 39.65 ± 11.55	ToM (WRT)	IL‐10 MDC	Positive correlation between plasma IL‐10 and MDC levels and ToM impairment
Ntouros et al. (2018)	DSM‐IV‐TR diagnostic criteria for SZ	12 UHR 25 FEP 16 SZ 23 HC	UHR: 24.5 ± 3.1 FEP: 25.48 ± 5.41 SZ: 35.56 ± 5.56 HC: 27.04 ± 2.91	ToM (PESIT) FER (PESIT)	IL‐10 TNF‐α TNF‐β IL‐1β IL‐8 IL‐4 Cortisol IGF‐1 IL‐2 IL‐5 IL‐12	UHR: Negative correlation between plasma IL‐4 levels and ToM impairment SZ: Negative correlation between plasma IL‐4 levels and FER impairment
King et al. (2021)	DSM‐IV‐TR diagnostic criteria for SZ or SZA	104 SZ/SZA 207 HC	SZ: 42 ± 10.95 HC: 36 ± 12.38	ToM (RMET, HTT) FER (ERT)	IL‐6	Positive correlation between plasma IL‐6 levels and ToM impairment Positive correlation between plasma IL‐6 levels and FER impairment
Mothersill et al. (2021)	DSM‐IV‐TR diagnostic criteria for SZ or SZA	53 SZ/SZA 207 HC	SZ/SZA: 42.55 ± 11.289 HC: 35.98 ± 12.032	FER (Paus Emotion Recognition Test)	IL‐6	Positive correlation between plasma IL‐6 levels and FER impairment Elevated plasma IL‐6 levels predicted an impaired neuronal response in brain regions (DMN) involved in facial emotion recognition in SZ/SZA patients compared with HC
Ospina et al. (2022)	DSM‐IV‐TR diagnostic criteria for SSD or other psychotic disorders Maintenance treatment with antipsychotics during 4 weeks	32 SZ	37 ± 10.0	FER (MSCEIT)	IL‐6 TNF‐α IL‐1β IL‐12p70 Eosinophil blood count	Negative correlation between plasma IL‐12p70 levels and FER impairment Negative correlation between plasma levels of eosinophils and FER impairment
King et al. (2022)	DSM‐IV diagnostic criteria for SZ or SZA	51 SZ/SZA 176 HC	SZ/SZA: 42 ± 11.25 HC: 36 ± 12.28	ToM (ToM cartoon task)	IL‐6	Positive correlation between plasma IL‐6 levels and ToM impairment Higher plasma IL‐6 levels predicted weaker suppression of the DMN during the Tom task
Li et al. (2022)	DSM‐IV diagnostic criteria for SZ Steady dose of antipsychotics for at least 6 months	44 SZ 59 HC	SZ: 47.02 ± 10.87 HC: 43.54 ± 11.38	FER (MSCEIT)	IL‐1β	Positive correlation between plasma IL‐1β levels and FER impairment
Pan et al. (2022)	DSM‐IV diagnostic criteria for SZ	75 SCZ 44 HC	SZ: 28.61 ± 6.90 HC: 30.07 ± 7.49	FER (MSCEIT)	TGF‐ β1	Positive correlation between plasma TGF‐ β1 levels and FER impairment
Baek et al. (2022)	DSM‐5 diagnostic criteria for SZ or other psychotic disorders Duration of illness < 1 year	111 SZ 6 SZA 47 schizophreniform disorder 10 specified and unspecified SSD	24 ± 4.5	ToM (False Belief Task, Picture story Task)	IL‐6 IL‐10 TNF‐α IFN‐γ IL 8 IL‐12 IL‐1β	Positive correlation between plasma IFN‐γ and IL‐12 levels and ToM impairment Positive correlation between plasma IL‐1β levels and ToM impairment
King et al. (2023)	DSM‐IV diagnostic criteria for SZ or SZA	53 SZ/SZA 176 HC	SZ/SZA: 42 ± 11.2 HC: 36 ± 12.2	FER (Paus Emotion Recognition Test)	IL‐6 IL‐8 IL‐10 TNF‐α	Positive correlation between plasma IL‐6 levels and FER impairment Higher plasma IL‐6 levels predicted weaker suppression of the DMN during face processing task

Abbreviations: CTQ, Childhood Trauma Questionnaire; DMN, default mode network; DSM‐IV‐TR, Diagnostic and Statistical Manual of Mental Disorders, Fourth edition (text revision); ERT, Emotion Recognition Task; FEP, first‐episode psychosis; FER, facial emotion recognition; fMRI, functional magnetic resonance imaging; HC, healthy controls; HTT, Hinting Task Test; IFN, interferon; IGF, insulin‐like growth factor; IL, interleukin; IL‐1RA, interleukin‐1 receptor antagonist; MCCB, MATRICS Consensus Cognitive Battery; MDC, macrophage‐derived chemokine; MSCEIT, Mayer–Salovey–Caruso Emotional Intelligence Test; PESIT, Perception of Social Inference Test; RMET, Reading in the Mind Eyes Test; sIL‐2R, soluble interleukin‐2 receptor; SZ, schizophrenia; SZA, schizoaffective disorder; TGF‐ β1, transforming growth factor β1; TNF, tumor necrosis factor; ToM, theory of mind; UHR, ultra‐high risk for psychosis group; WRT, Waiting Room Task.

### 3.1 Inflammatory Markers and ToM Impairment

Associations between ToM and inflammatory markers in patients with schizophrenia were found in several studies. Four studies explored the association between plasma IL‐10 levels and ToM impairment (Baek et al. 2022; Dunne et al. 2017; Ntouros et al. 2018; Walss‐Bass et al. 2013). Among these studies, two of them reported a positive correlation (Dunne et al. 2017; Walss‐Bass et al. 2013).

Additionally, the association between plasma IL‐1β levels and ToM impairment was assessed in three studies (Baek et al. 2022; Ntouros et al. 2018; Walss‐Bass et al. 2013), where two of them found a positive correlation between these two variables (Baek et al. 2022; Walss‐Bass et al. 2013). The association between plasma IL‐6 levels and ToM impairment was studied by four authors (Baek et al. 2022; King et al. 2021, 2022; Walss‐Bass et al. 2013). Two studies identified a positive correlation between these two variables (King et al. 2021, 2022), whereas the other two studies found no significant association (Baek et al. 2022; Walss‐Bass et al. 2013). Moreover, none of the selected studies reported an association between plasma TNF‐α levels and ToM impairment (Baek et al. 2022; King et al. 2023; Ntouros et al. 2018; Ospina et al. 2022; Walss‐Bass et al. 2013).

Furthermore, the association between plasma IL‐12 levels and ToM impairment was investigated in two studies (Baek et al. 2022; Ntouros et al. 2018), one of which identified a positive correlation between these variables (Baek et al. 2022). This same study also found a positive correlation between plasma IFN‐γ levels and ToM impairment. However, two other studies based on the plasma levels of these two cytokines reported no significant association with ToM impairment (Ntouros et al. 2018; Walss‐Bass et al. 2013). Walss‐Bass and colleagues identified a positive correlation between plasma IL1‐RA and sIL‐2R levels and ToM impairment (Walss‐Bass et al. 2013), whereas Dunne and colleagues found a positive correlation between plasma MDC levels and ToM impairment (Dunne et al. 2017). Conversely, Ntouros and colleagues reported a negative correlation between plasma IL‐4 levels and ToM impairment (Ntouros et al. 2018).

Associations of other inflammatory markers, such as IL‐2, IL‐5, IGF‐1, IL‐8, cortisol, and TNF‐β, with ToM impairments were not reported.

### 3.2 Inflammatory Markers and FER Impairment

The association between FER impairment and inflammatory markers in patients with schizophrenia was assessed in seven studies (King et al. 2021 ; King et al. 2023; Li et al. 2022; Mothersill et al. 2021; Ntouros et al. 2018; Ospina et al. 2022; Pan et al. 2022). To our best knowledge, there are no studies having explored vocal emotion recognition (emotional prosody) or emotional expression impairment.

Among the studies based on FER assessment, four studies explored the association between plasma IL‐6 levels and FER impairment (King et al. 2021; King et al. 2023; Mothersill et al. 2021; Ospina et al. 2022). A positive correlation between these two variables was found in three studies, which included fMRI during a dynamic emotion recognition task (King et al. 2021; King et al. 2023; Mothersill et al. 2021). These studies reported that high plasma levels of IL‐6 predicted an altered neuronal response in the Default ode Network, brain regions involved in social cognition and in particular in FER. Conversely, Ospina and colleagues reported no association between these variables, but they found a negative correlation between plasma IL‐12p70 and eosinophil levels, and FER impairment (Ospina et al. 2022). Additionally, Ntouros and colleagues identified a negative correlation between plasma IL‐4 levels and FER impairment (Ntouros et al. 2018). Moreover, the association of plasma IL‐1β levels and FER impairment was assessed by three authors (Li et al. 2022; Ntouros et al. 2018; Ospina et al. 2022), and only one found a positive correlation between these two variables (Li et al. 2022). Finally, a positive correlation between TGF‐ β1 and FER impairment was found in one study (Pan et al. 2022).

Associations of other inflammatory markers, such as IL‐10, TNF‐α and TNF‐β, IL‐8, cortisol, IGF‐1, IL‐12, IL‐5, and IL‐2, with FER impairment were not reported.

None of the included studies explored the link between inflammatory markers and attributional style, social knowledge, and social cue perception in patients with SSD.

## 4 Discussion

The aim of our systematic review was to precise the relationships between inflammatory markers and social cognitive impairments in patients with SSD. Eleven studies were included, primarily focusing on the domains of ToM and FER. These two aspects of social cognition are fundamental to interpersonal functioning and are known to significantly impact the functional outcomes of patients with SSD (Halverson et al. 2019). Indeed, some authors suggest that higher plasma levels of cytokines predict social functioning impairments (Garés‐Caballer et al. 2022; González‐Blanco et al. 2019).

Our findings provide evidence supporting the hypothesis that inflammation plays a role in social cognitive deficits in SSD. Among the inflammatory markers studied, IL‐6 was the most frequently examined and demonstrated consistent positive correlations with both ToM and FER impairments (King et al. 2021, 2022; King et al. 2023; Mothersill et al. 2021). IL‐6 contributes to neuroinflammation by activating microglial cells and disrupting neural connectivity in key brain networks such as the default mode network (DMN), which are critical for social cognition abilities (Kirkpatrick and Miller 2013; Li et al. 2014; Spies et al. 2017). Indeed, IL‐6 appears to affect both the connectivity of the DMN at rest and during social cognitive tasks (Anticevic et al. 2012; Fornito et al. 2012; King et al. 2023; Zhou et al. 2016). Moreover, IL‐6 can affect the activity of the insula and medial prefrontal cortex, two key regions for emotion processing (Mothersill et al. 2021). Finally, L‐6 appears to mediate the relationship between childhood trauma, a known risk factor for social cognitive impairments, and social cognitive abilities (King et al. 2021, 2022; King et al. 2023). This suggests that IL‐6‐induced inflammation may be one mechanism by which early stress experiences contribute to the development of social cognitive problems later in life. However, three studies did not find a correlation between plasma IL‐6 levels and social cognitive impairments probably due to small sample sizes and no control group, which limited the interpretation of the results (Baek et al. 2022; Ospina et al. 2022; Walss‐Bass et al. 2013). Moreover, these studies did not make multiple comparisons to assess the impact of potentially confounding variables on plasma IL‐6 levels. Our results reinforce the hypothesis already reported in several reviews and meta‐analyses that IL‐6 is the main cytokine that can be considered as a trait factor in schizophrenia (Çakici et al. 2019; Goldsmith et al. 2016; Miller et al. 2011), but further studies are needed to refine the role of IL‐6 on social cognition in patients with SSD.

IL‐1β is the second most cytokine explored in the selected studies, where three studies have found a significant positive correlation with ToM and FER impairments (Baek et al. 2022; Li et al. 2022; Walss‐Bass et al. 2013). Previous studies had already highlighted the existence of correlations between plasma IL‐1β levels in patients with SSD (Dawidowski et al. 2021; Halstead et al. 2023; Momtazmanesh et al. 2019; Zhu et al. 2018). In addition, IL‐1β would have a negative impact on hippocampal circuits involved in neurocognition such as memory and cognitive flexibility, but also in FER, empathy, and social interactions (Banker et al. 2021; Immordino‐Yang and Singh 2013; Perry et al. 2011; Rubin et al. 2014). Two studies in our review found no correlation likely due to small samples and group heterogeneity (Ntouros et al. 2018; Ospina et al. 2022).

Concerning IL‐10, our results were contradictory with two studies finding a positive correlation between plasma IL‐10 levels and ToM impairments (Dunne et al. 2017; Walss‐Bass et al. 2013); the other three studies found no correlation between plasma IL‐10 levels and ToM or FER impairments (Baek et al. 2022; King et al. 2023; Ntouros et al. 2018). Previous studies also showed mixed results with some studies reporting lower IL‐10 plasma levels in acutely relapsed patients with schizophrenia (Goldsmith et al. 2016; Miller et al. 2011) and patients with ultra‐high risk of psychosis (Park and Miller 2020) compared to control subjects contradictory with a meta‐analysis who found no significant correlation between plasma IL‐10 levels and risk of psychotic transition (Misiak et al. 2021). Additionally, Leboyer and colleagues reported that plasma IL‐10 levels would be significantly higher in patients with treatment‐resistant schizophrenia compared to patients with nonresistant schizophrenia (Leboyer et al. 2021). In summary, these conflicting results could be explained by variations in study designs, cytokine measurement techniques, or by differences among patients, stage of disease, and treatments received. These mixed findings highlight the complex role of IL‐10 in schizophrenia and the need for further research to clarify its effects.

Four studies measured IL‐8 plasma levels, finding no correlation with ToM or FER impairments identified (Baek et al. 2022; King et al. 2023; Ntouros et al. 2018; Walss‐Bass et al. 2013). While previous studies reported elevated IL‐8 plasma levels in patients with untreated first‐episode of psychosis or schizophrenia compared with control subjects (Çakici et al. 2020; Halstead et al. 2023; Miller et al. 2011; Wang and Miller 2018), a recent meta‐analysis found no difference between patients with schizophrenia and healthy controls subjects (Pillinger et al. 2019). None of them has investigated the relationship between cognitive impairments and plasma IL‐8 levels in patients with schizophrenia. However, Willette et al., in an animal study, found that high levels of IL‐8 were associated with a decrease in hippocampal gray matter, a region associated with neurocognition and social cognition (Willette et al. 2013).

Regarding the cytokine TNF‐α, five studies measured this inflammatory marker (Baek et al. 2022; King et al. 2023; Ntouros et al. 2018; Ospina et al. 2022; Walss‐Bass et al. 2013) but none of them found a correlation with ToM or FER impairments. This observation could be explained by the clinical symptoms' heterogeneity of the population. Previous studies have reported significantly higher plasma TNF‐α levels in patients with acute schizophrenia with agitation or depressive symptoms, whereas patients with chronic schizophrenia presented lower plasma TNF‐α levels compared to healthy controls (Halstead et al. 2023; Lee et al. 2017; Lv et al. 2015; Wang et al. 2024). Moreover, according to a review, there is a negative correlation between plasma TNF‐α levels and neurocognitive impairments in patients with schizophrenia compared with control subjects (Momtazmanesh et al. 2019). TNF‐ β was measured in one study, which identified no significant correlation between plasma levels of this variable and ToM or FER impairments (Ntouros et al. 2018).

Furthermore, sIL‐2R, IL‐1RA, and IFN‐γ plasma levels were measured in one study, showing a positive correlation with ToM impairments. A recent meta‐analysis found elevated IL‐1RA and sIL‐2R in acute and chronic schizophrenia, while IFN‐γ was elevated in acute and reduced in chronic SSD (Halstead et al. 2023). Moreover, Leboyer and colleagues reported significantly higher plasma IFN‐γ levels in patients with treatment‐resistant or ultra treatment–resistant schizophrenia compared to patients with nonresistant schizophrenia (Leboyer et al. 2021). However, IFN‐γ seemed to be a promising lead as a possible specific marker of social cognition (Moieni et al. 2015; Monteiro et al. 2016). IFNγ would seem to act as a negative regulator of hippocampal function, affecting cognition and potentially social cognition. Its absence enhances hippocampal neurogenesis, dendritic growth, and cognitive performance in learning and memory tasks. While its role in social cognition remains underexplored, IFNγ’s influence on inflammation‐related cognitive dysfunction suggests that it may contribute to social cognition impairments observed in inflammatory contexts. Targeting IFNγ signaling could offer therapeutic opportunities (Monteiro et al. 2016).

Additionally, plasma IL‐12 levels were measured in three studies (Baek et al. 2022; Ntouros et al. 2018; Ospina et al. 2022), including its active heterodimer IL‐12p70. One showed a negative correlation between IL‐12p70 and FER impairments, while another found a positive correlation between IL‐12 and ToM impairments (Baek et al. 2022; Ospina et al. 2022). The same study reported a negative correlation between eosinophil levels and FER impairments in patients with schizophrenia (Ospina et al. 2022). These markers do not appear to be specific to social cognition or to patients with SSD. Indeed, several authors have found elevated plasma levels of IL‐12 and eosinophils in patients with depression (Osimo et al. 2020; Singh et al. 2022), and these patients also have FER impairments (Krause et al. 2021). Further research is needed to confirm the role of these markers in FER impairments.

Moreover, plasma IL‐4 levels were measured in one study, which identified a negative correlation between ToM and FER impairments in patients with SSD (Ntouros et al. 2018). These findings seem to be in agreement with previous studies, which report a probable neuroprotective effect of IL‐4 in various pathologies and mainly on neurocognitive functions (Dionisio‐Santos et al. 2020).

Furthermore, one of the included studies found a positive correlation between plasma MDC levels and ToM impairments (Dunne et al. 2017). While MDC and peripheral chemokine levels are less studied than nonchemokine cytokines, elevated MDC levels were reported in both patients with first episode of psychosis or schizophrenia compared to healthy controls. These findings suggest that higher levels of MDC may be linked to an increased sensitivity to negative social cues, which could contribute to difficulties in social interaction in these patients. Interestingly, this correlation was not observed in delusional patients, highlighting the possibility of different immune mechanisms at play in schizophrenia subtypes. Furthermore, Dunne and colleagues found that MDC and IL‐10 were not associated with any measures of pure neurocognition or psychiatric symptoms, indicating that these cytokines may be specifically linked to social cognitive impairments. However, further research is needed to confirm these findings and determine the precise mechanisms by which MDC influences social cognition in SSD.

Finally, one of the included studies found a positive correlation between plasma TGF‐ β1 levels and FER impairments (Pan et al. 2022). Although several studies have found elevated plasma levels of TGF‐β in patients with SSD, including those with acute relapses and first‐episode psychosis (Kim et al. 2004; KÖŞger et al. 2020; Miller et al. 2011), these results are contradictory with a recent study of Raschick and colleagues. It was found that elevated levels of TGF‐β1 contribute to better preservation of hippocampal structures, particularly the dentate gyrus, which may be involved in cognitive functions (Raschick et al. 2023).

None of the studies included in our review have explored the relationship between inflammatory markers and attributional style or social perception in patients with SSD. To our knowledge, no study has explored the association between attributional style or social perception and inflammatory markers in the general population or in other neuropsychiatric disorders. The lack of results could be explained by two meta‐analyses that have reported no significant difference in attributional style between patients with schizophrenia and control subjects (Savla et al. 2013; Weinreb et al. 2022) and because social perception as a cognitive function remains under investigated.

The systematic review presents several limitations. First, the number of studies was small with a limited overall sample size, and the number of participants included in the different studies was not homogeneous. For example, patients with schizoaffective disorder generally demonstrate better social cognitive abilities compared to those with schizophrenia, particularly in understanding others’ mental states and processing facial information. While both groups may struggle with FER, patients with schizophrenia tend to have greater impairments regarding emotions such as fear and happiness. The brain mechanisms underlying facial information processing appear to be more impaired in patients with schizophrenia, whereas the mood disturbances characteristic of patients with schizoaffective disorder do not significantly disrupt this ability. These differences highlight a relative preservation of social cognitive abilities in patients with schizoaffective disorder, especially regarding ToM and emotion recognition (Chen et al. 2006; Fiszdon et al. 2007).

Two studies did not include a group of healthy controls, which reduced their quality (Baek et al. 2022; Ospina et al. 2022). Moreover, key clinical variables like age of onset, illness course, and treatment history were not consistently considered.

In addition, the cross‐sectional design of the included studies limits causal conclusions, and longitudinal studies are needed to establish causality between biomarkers and cognitive impairments.

Finally, a significant limitation is a disproportionate focus in literature on the relationship between inflammatory markers and neurocognition (Dawidowski et al. 2021; Halstead et al. 2023; Laurikainen et al. 2020; Momtazmanesh et al. 2019; Peng et al. 2022; Sahoo et al. 2023; Zhu et al. 2018), with far less attention given to social cognition. Moreover, very few studies utilize comprehensive test batteries that evaluate both neurocognition and social cognition while examining their connections to inflammation. This gap is surprising, given the well‐established role of neurocognition as a mediator of social cognition (Hoe et al. 2012). Addressing this limitation is essential for a more integrated understanding of the interplay between inflammation, neurocognitive functions, and social cognitive processes.

## 5 Conclusion

Although the correlations do not seem to be specific to social cognitive abilities, they reinforce the hypothesis of a correlation between inflammatory markers and cognition in SSD with notable differences according to the course of illness. Many studies have found significant correlations between IL‐6 plasma levels and cognitive disorders, allowing us to assume that IL‐6 plasma levels could be a factor in subgroups of patients with schizophrenia (Halstead et al. 2023; Miller et al. 2011). On the other hand, the changes in inflammatory markers plasma levels found in patients with schizophrenia could be related to mutations in the genes encoding them (Butler et al. 2016; Frydecka et al. 2015). It may, therefore, be relevant in the future to explore whether there are specific genetic abnormalities in cognitive impairments common to inflammatory markers in certain subgroups.

These results encourage the development of precision psychiatry to offer individualized and personalized treatment strategies for subgroups of patients. Patient subgroups should be characterized according to their clinical presentation (predominant types of symptoms and severity of them) and their biotypes (cytokine profile notably) to offer precision and comprehensive cares (Clementz et al. 2022). For example, the ability to identify patients with high levels of specific biomarkers such as IL‐6 and cytokine gene polymorphisms could lead to personalized immunotherapy.

Beyond more specific therapies, antipsychotic treatment, widely used in patients with schizophrenia, is also thought to play an anti‐inflammatory role (Ferrari et al. 2023; Juncal‐Ruiz et al. 2018; Røge et al. 2012; Sobiś et al. 2015), whereas other nonpsychotropic treatments with an inflammation modulating effect seem promising as monotherapy or adjuvant treatment in patients with schizophrenia, such as minocycline, N‐acetylcysteine, NSAIDs, omega‐3 fatty acids and davunetide, pregnenolone, tocilizumab, and memantine (Amminger et al. 2010; Arvindakshan et al. 2003; Chaudhry et al. 2012; Farokhnia et al. 2013; Girgis et al. 2018; Javitt et al. 2012; Liu et al. 2014; Marx et al. 2011; Murugan et al. 2019; Nitta et al. 2013; Zheng et al. 2018).

## Author Contributions

**Alexandre Carpentier**: conceptualization, methodology, formal analysis, visualization, writing–original draft, writing–review and editing. **Dimitrios Zampetas**: writing–original draft. **Alexandre Durand**: conceptualization, methodology, visualization. **Mickaël Naassila**: visualization, writing–review and editing, supervision. **Marie‐Cécile Bralet**: conceptualization, methodology, validation, writing–review and editing, visualization, supervision.

## Conflicts of Interest

Marie‐Cécile Bralet has received honoraria for lectures and nonfinancial support from Boehringer, Otsuka, and Lundbeck, outside the submitted work. All other authors declare no conflicts of interest.

### Peer Review

The peer review history for this article is available at https://publons.com/publon/10.1002/brb3.70384.

## Data Availability

This study is a systematic review, and no new data were generated. All data supporting the findings of this review are available from the original studies cited in the reference list
